# Correlates of Depressive Symptoms in Older Adults with Diabetes

**DOI:** 10.1155/2016/8702730

**Published:** 2015-11-22

**Authors:** LaRita C. Jones, Olivio J. Clay, Fernando Ovalle, Andrea Cherrington, Michael Crowe

**Affiliations:** ^1^Department of Psychology, University of Alabama at Birmingham, Birmingham, AL 35294, USA; ^2^Diabetes & Endocrine Clinical Research Unit, Division of Endocrinology, Diabetes and Metabolism, University of Alabama at Birmingham, Birmingham, AL 35294, USA; ^3^Division of Preventive Medicine, University of Alabama at Birmingham, Birmingham, AL 35294, USA

## Abstract

Investigators examined correlates of depressive symptoms within a sample of older adults with diabetes. Participants completed a structured telephone interview with measures including depressive symptoms, health conditions, cognitive function, and diabetes distress. Correlations and hierarchical linear regression models were utilized to examine bivariate and covariate-adjusted correlates of depressive symptoms. The sample included 246 community-dwelling adults with diabetes (≥65 years old). In bivariate analyses, African Americans, individuals with specific health issues (neuropathy, stroke, respiratory issues, arthritis, and cardiac issues), and those with higher levels of diabetes distress reported more depressive symptoms. Older age, higher education, more income, and better cognitive function were inversely associated with depressive symptoms. In the final covariate-adjusted regression model, stroke (*B* = .22, *p* < .001), cognitive function (*B* = −.14, *p* < .01), and higher levels of diabetes-related distress (*B* = .49, *p* < .001) each were uniquely associated with more depressive symptoms. Diabetes distress partially mediated the associations between cardiac issues and depressive symptoms and between cognitive function and depressive symptoms. Findings suggest that interventions targeted at helping older adults manage their diabetes-related distress and reducing the likelihood of experiencing additional health complications may reduce depressive symptoms within this population.

## 1. Introduction

The current impact of diabetes at individual and societal levels in the USA is substantial. According to the Centers for Disease Control and Prevention (CDC), approximately 29.1 million individuals in the USA (9.3% of the population) are currently living with diabetes [1]. The CDC estimates that 25.9% of Americans aging 65 or older have this chronic illness. On average, individuals with diabetes spend more than 2.3 times the amount in healthcare expenses than those without diabetes [1]. There is a clear need to do a better job of preventing diabetes at early ages as well as manage diabetes and comorbid conditions in older adults.

The combination of financial, physical, and mental demands due to living with a chronic illness such as diabetes can result in emotional distress and depressive symptoms. Due to the increasing prevalence of older individuals who are living with chronic illness, there is a need for researchers to investigate and better understand the various causes of depressive symptoms in this population. In order to reduce and better manage stressors, one must first have a general understanding of how stressors in the context of chronic illness are processed both emotionally and physically.

### 1.1. Biopsychosocial Model

In 1977, George Engel proposed the biopsychosocial cultural model (later shortened to “biopsychosocial model” for the sake of brevity). Engel proposed this model under the hypothesis that to provide a basis for understanding what influences the trajectory of a disease (for treatment and prevention purposes) we must not only examine the biological factors affecting the individual, but also examine the social context in which that individual lives [2]. With this theory, the fields of medicine and research gained a better understanding of the relationships between suffering, disease, and illness as well as a clearer understanding of an individual's subjective experience and the influence it can have on diagnoses, health outcomes, and overall healthcare [3]. It is with an understanding of the biopsychosocial model that researchers of the current study sought to examine depressive symptoms and diabetes distress in a sample of older adults living with diabetes.

### 1.2. Stressors and Mental Health

The relationship between the presence of health conditions such as stroke, cardiovascular disease, respiratory issues, arthritis, and neuropathy and individuals reporting depressive symptoms has been well documented [4–7]. Previous research also suggests that experiencing the pain of neuropathy coupled with the possible decrease in independence may have a negative impact on mood [8]. For individuals who are coping with the effects of a stroke and are also dealing with cognitive function difficulties that often follow a stroke, in addition to the day-to-day management of their diabetes, psychological distress may be exacerbated [9]. It has also been proposed that respiratory issues, such as asthma, and negative moods have a mutually potentiating relationship [10]. It appears that an individual's heart health and the ability to control chronic health issues are very closely related to mood problems [11]. Furthermore, depressive symptoms are more prevalent in older individuals who experience arthritic pain [12]. In addition to the relationship with health conditions, the prevalence of depressive symptoms seems to be considerably higher among older individuals who are of lower educational attainment, are female, and/or are minorities [13–15].

#### 1.2.1. Cognitive Function, Diabetes, and Depressive Symptoms

Depressive symptoms are often exhibited at the onset or during the early manifestations of cognitive decline and dementia [16, 17]. Decline in cognitive function is an issue particularly important for older individuals who experience diabetes. For example, in a longitudinal study of 624 community-dwelling older adults, it was found that having diabetes predicted cognitive decline, particularly among individuals with less frequent physician visits and African Americans who reported higher levels of perceived discrimination [18].

#### 1.2.2. The Role of Diabetes Distress

Diabetes distress is the term used to describe the emotional strains that are commonly associated with living with diabetes. Fisher and colleagues examined associations between major depression, depressive symptoms, diabetes distress, and glycemic control [19]. Diabetes distress was found to differ from both clinical depression and depressive symptoms so far as it was more closely tied to poorer glycemic control and disease management. Their findings lend support to the idea that different methods of treatment focusing specifically on health-related distress may be particularly beneficial for improving depressive symptoms in this population [19, 20].

The purpose of this study was to examine correlates of depressive symptoms in older adults with diabetes and the extent to which diabetes distress may explain associations between depressive symptoms and factors that have been previously linked to these symptoms. It was hypothesized that older individuals with diabetes who reported additional adverse medical conditions would also report a higher number of depressive symptoms. Among older individuals living with diabetes, lower cognitive function was hypothesized to be associated with more depressive symptoms. Finally, reporting high levels of diabetes-related distress was hypothesized to be associated with high levels of depressive symptoms and to potentially explain associations between health, cognitive function, and depressive symptoms.

## 2. Methods

Data comes from the Diabetes and Aging Study of Health (DASH). The DASH sample included community-dwelling older adults in the Birmingham, Alabama area, as well as patients from a diabetes clinic at the University of Alabama at Birmingham (UAB). All participants were required to be 65 years of age or older and identified as having diabetes via either self-report or physician diagnosis. Community-dwelling participants were recruited from a commercially available list of older adults in the Birmingham metropolitan area that is maintained by the UAB Roybal Center for Translational Research on Aging and Mobility. Clinic participants were recruited from patients of one physician at the UAB Diabetes & Endocrinology Clinic. All participants were contacted via a mailed letter followed by telephone contact. African Americans were oversampled in order to fulfill DASH's overarching goal, examining racial disparities in mental health, cognitive function, and mobility outcomes in older adults with diabetes over time. Participants completed telephone interviews focused on diabetes-specific measures of health and psychosocial factors as well as performance-based cognitive testing.

### 2.1. Demographics

Demographic variables were gathered via self-report. Age, race, gender, years of education, marital status, and income were obtained. Income was measured in ordinal categories ranging from 1 (less than $5,000) to 9 ($100,000 or greater).

### 2.2. Health Problems

Health issues were assessed via self-report. A list of health issues that commonly occur in older adults was assessed, and participants reported if they had ever been told by a doctor/nurse that they have had various health conditions including neuropathy, stroke (or a “ministroke”/transient ischemic attack), respiratory issues (asthma, chronic bronchitis, or emphysema), arthritis, and cardiovascular problems (heart attack/myocardial infarction or congestive heart failure). Responses were coded as 0 (no) and 1 (yes).

### 2.3. Cognitive Functioning

Cognitive functioning was assessed using the modified Telephone Interview for Cognitive Status (TICS-M) which measures global cognitive status in older adults [21]. This is a 13-item modified and previously validated version of the Telephone Interview of Cognitive Status. The TICS-M includes four domains: orientation; registration/recent memory and delayed recall; attention/calculation; and semantic memory, comprehension, and repetition. The possible range of scores is 0 to 39, with a relatively higher proportion of the total score being allocated to the memory component [21]. A score of 20 or lower suggests cognitive impairment.

### 2.4. Depressive Symptoms

Mental health was assessed via the Geriatric Depression Scale-Short Form. This scale is comprised of 15 items which assess low moods and feelings of helplessness (common symptoms of depression) in the individual [22]. The potential range of this scale is from 0 to 15 and Cronbach's alpha is 0.85. A score of 5 or higher suggests elevated depressive symptoms [23].

### 2.5. Diabetes Distress

The scale used to measure diabetes-related emotional distress was a brief 2-item version [24] that was modified from the original 17-item Diabetes Distress Screening Scale (DDSS) [25]. For the DDSS2, participants were asked to rate how much the following items caused distress during the past month: “Feeling overwhelmed by the demands of living with diabetes,” and “Feeling that I am often failing with my diabetes routine.” Response options range from 1 (indicating the item was not a problem) to 6 (indicating that the item was a very serious problem). Cronbach's alpha for the two items was 0.79. A summary score was created for diabetes distress with a sum of 6 or greater corresponding to “moderate distress” and potentially identifying individuals who are at-risk for negative outcomes.

### 2.6. Analyses

Analyses were conducted using SAS V9.1.3 [26] and IBM SPSS Version 22 [27]. Bivariate correlations between depressive symptoms and variables of interest were examined. An initial regression model included demographic characteristics (age, gender, education, marital status, income, and race) as correlates of depressive symptoms. Subsequent models examined associations between depressive symptoms and variables categorized as potential problems for older adults with diabetes by adding health conditions (neuropathy, stroke, respiratory issues, arthritis, and cardiac issues), cognitive function, and diabetes distress in additional sequential models. In order to determine the contribution of additional variables to the regression model, an increment *r*-square test was assessed after each step.

## 3. Results

### 3.1. Participants

Descriptive characteristics for participants are presented in Table 1. There were 246 participants (172 community-based and 74 clinic-based ones), with 126 (51.44%) Caucasians, 110 (44.72%) African Americans (AA), and 10 “other” races (4.07%). There were 109 male participants (44.31%), and the average age was 73.35 years (range 65–90). Their self-reported time since diagnosis ranged from less than a year to 58 years, with an average of 16 years. In the 246 participants, the most frequent health condition was arthritis (71.54%). The average sample score on the TICS-M was 23.37 (SD = 5.59), slightly above the suggested cutoff for cognitive impairment of 21. Sixty-five participants (26.42%) scored 20 or less on the TICS-M, indicating that they experienced problems with cognitive function. In terms of diabetes-related distress, the average score on the DDSS2 was 3.85 (SD = 2.51). Forty-eight participants (19.51%) scored at or above the cut-off score of 6 indicating that they were experiencing moderate to severe distress. The average score for the sample on the GDS (cut-off score of 5 or higher for clinically significant symptoms) was 2.73 (SD = 2.96). Forty-three participants (17.48%) scored at or above this cutoff, indicating that they experienced an elevation in depressive symptoms.

Correlations are presented in Table 2. These results indicated that African Americans reported more depressive symptoms than Caucasians (*r* = .17, *p* < .01). Participants who were older (*r* = −.16, *p* = .01) had higher income (*r* = −.18, *p* < .001), and more education (*r* = −.22, *p* < .001) reported fewer depressive symptoms. Regarding the specific health conditions of interest, people with neuropathy (*r* = .21, *p* < .01), stroke (*r* = .36, *p* < .0001), respiratory issues (*r* = .20, *p* < .01), arthritis (*r* = .17, *p* < .05), and cardiac issues (*r* = .23, *p* < .001) also experienced greater levels of depressive symptoms. Additionally, those within the sample who had better cognitive function (*r* = −.27, *p* < .001) reported fewer depressive symptoms.

Regarding correlations between diabetes distress and other variables of interest (Table 2), those individuals who experienced more depressive symptoms also experienced more diabetes distress (*r* = .60, *p* < .0001). Lower levels of diabetes distress were also seen in those with more years of education (*r* = −.18, *p* < .01), more income (*r* = −.23, *p* < .001), and married individuals (*r* = −.13, *p* < .05). African American participants indicated more diabetes distress than Caucasians (*r* = .19, *p* < .01). Participants who experienced neuropathy (*r* = .16, *p* = .01) and cardiac issues (*r* = .17, *p* < .01) also reported greater levels of diabetes distress. Finally, those individuals with better cognitive function indicated that they had less diabetes distress (*r* = −.18, *p* < .01).

### 3.2. Hierarchical Regression Models

The association between demographic factors and depressive symptoms was initially examined (Table 3). There was a significant covariate-adjusted relationship between age and depressive symptoms, indicating that older age was associated with fewer depressive symptoms (*B* = −.08, *p* < .05). Additionally, the significant association between education and depressive symptoms indicated that being more educated was associated with fewer depressive symptoms (*B* = −.17, *p* < .05). None of the other demographic factors were significantly related to depressive symptoms in this covariate-adjusted model.

Adding health problems and cognitive function to the model accounted for a significant amount of variance above and beyond the variability accounted for by demographic variables alone: *R* ^2^Δ = .212, *p* < .001 (Table 3, Model 2). In this model, the association between older age and fewer depressive symptoms remained significant. A number of health problems had unique associations with more depressive symptoms: neuropathy and arthritis: *p*'s <.05; cardiac issues: *p* < .01; and stroke: *p* < .0001. Additionally, higher levels of cognitive function were associated with fewer depressive symptoms (*p* < .01).

In the final model, adding diabetes distress accounted for a significant amount of variance above and beyond the variability accounted for by demographic variables, health issues, and cognitive function: *R* ^2^Δ = .20, *p* < .001 (Table 3, Model 3). Although diabetes distress and depressive symptoms were moderately correlated, an examination of the variance inflation factor (1.19) and tolerance (0.84) provided evidence that multicollinearity would not be a concern when interpreting the findings. Of the variables examined in the final model, stroke (*B* = .20, *p* < .001) and experiencing diabetes distress (*B* = .49, *p* < .001) had the strongest associations with depressive symptoms. The associations between neuropathy and depressive symptoms and between respiratory issues and depressive symptoms remained statistically significant. The relationship between cardiac issues and depressive symptoms was mediated by diabetes distress, and the relationship between cognition and depressive symptoms was partially mediated by diabetes distress. Individuals with cardiac issues and those with lower levels of cognitive function reported more diabetes distress, and higher levels of diabetes distress were, in turn, associated with reporting more depressive symptoms. The relationship between cardiac issues and cognitive function was reduced by 47.01% after adding diabetes distress to the model and the association between cognitive function and depressive symptoms was reduced by 26.80%.

## 4. Discussion

This study was conducted to examine correlates of depressive symptoms in older adults living with diabetes. The sample consisted of older African American and Caucasian individuals diagnosed with diabetes prior to their participation in the study. The results yielded several findings that are consistent with previous literature, as well as new findings that may provide insight into potential ways to reduce depressive symptoms within the growing population of older individuals with diabetes.

It was hypothesized that individuals who reported adverse medical conditions in addition to a diabetes diagnosis would report a higher number of depressive symptoms. Results of this investigation revealed that individuals who had suffered a stroke reported substantially more depressive symptoms than those with no history of stroke. The association between stroke and depressive symptoms in older individuals with diabetes is important to discern, as previous studies indicate that many of the adverse effects of stroke are associated with elevated depressive symptoms [7, 28]. For example, stroke is one of the leading causes of permanent mobility limitation in the US, and mobility limitation has been shown to be associated with elevated depressive symptoms in older adults [29]. Stroke is also one of the leading causes of cognitive impairment. The relationship between stroke, depressive symptoms, and cognitive impairment is still unclear: many scientists are investigating whether poststroke depressive symptoms and cognitive impairment develop exclusive of one another or if there is a mutually potentiating relationship between the two. Regardless of causation, this interrelationship has important implications for older individuals with diabetes, as individuals who live with diabetes are at an increased risk of suffering both stroke and impaired cognition [9, 30, 31].

Our examination of cognitive function yielded results similar to those from the existing literature as well. It is quite common for researchers and healthcare professionals to see high levels of depressive symptoms in individuals with cognitive function impairments. Supporting a possible bidirectional relationship, high levels of depressive symptoms are also predictive of greater cognitive decline [31]. Analyses from the current study indicated that cognitive function has a significant association with depressive symptoms, where higher cognition is linked to lower depressive symptoms.

While the significant association between cognitive function and depressive symptoms is an interesting finding that should be further explored longitudinally in older adults with diabetes, the association became nonsignificant and was substantially reduced after addition of diabetes distress to the model. Thus, it is possible that worse cognitive function may be linked to depressive symptoms due to greater difficulty in managing diabetes.

The robust association between diabetes distress and depressive symptoms suggests the possibility that in this context the GDS is identifying individuals with elevated diabetes-specific distress rather than (or in addition to) symptoms of depression per se, though without the inclusion of a structured clinical interview for depression we can not confirm whether or not this is the case. The results are, however, similar to those found in Fisher and colleagues' study [19], in which it was determined that higher scores on the scale being used to measure depressive symptoms appeared to reflect diabetes-specific distress. This is an important finding regarding diabetes and depressive symptom research, as the majority of distress treatment methods are based on literature for the treatment of depression [19]. It is possible that individuals with diabetes are not receiving the appropriate type of treatment for their elevated depressive symptoms, which may appear to be similar to those seen in clinical depression but may be unique to having diabetes itself. It is also important to note that diabetes distress could potentially be modifiable, and, due to the high correlation between diabetes distress and depressive symptoms, it is feasible that these symptoms may be decreased by lowering diabetes distress.

A limitation of the current study is that much of the data were collected via self-report. However, we utilized a widely used screening measure for depressive symptoms in older adults as well as a validated performance-based measure of cognitive performance. While there were efforts to interview participants while they were in a quiet setting without distractions, this could not always be guaranteed. For any research study using telephone interviews, being distracted by something or someone nearby could affect both the participants' abilities to answer questions as accurately as possible and willingness to answer some of the questions in general. Additionally, the telephone-based interview did not allow for in-person A1C collection. Although participants were asked to indicate their latest A1C test result, a large portion of participants lacked knowledge of their result. Therefore, A1C was not used as a measure of diabetes control.

Because this study did not have access to medical records, an important question is whether self-report of diabetes is accurate. Prior studies have found that reliability of self-reported diabetes is very high compared to information from general practitioners as well as medical record data [32, 33]. In one of these studies, researchers reported that cognitive function measured with the Mini-Mental State Examination (MMSE) was not associated with poorer accuracy of reporting diabetes or other specific chronic diseases [32]. The main problem with diabetes self-reporting is with false-negatives (individuals with diabetes who do not report having diabetes) rather than false-positives [33]. However, in the current study, only individuals who reported having diabetes were included. Still, we did not have information on potentially important factors such as diabetes control or mental health history prior to diabetes diagnosis, which may be relevant to understanding depressive symptoms in older age. It should also be noted that information regarding family history of depression and comorbid substance use disorders were not gathered. Substance use disorder comorbidity, in particular, could directly affect diabetes control as well as depressive symptoms.

Due to the cross-sectional design of this analysis, it is impossible to determine causality or to investigate possible bidirectional associations between variables of interest. Lastly, results from this study are not necessarily generalizable to all individuals living with diabetes: the majority of the sample was from the greater Birmingham area in Alabama, and all participants were aware that they had diabetes. Thus, findings from this study may be generalizable only to those with knowledge of their diabetes status. Also relevant to generalizability, there was good representation of older African Americans, who are at greater risk of negative diabetes-related outcomes [34] and comprised 45% of the current sample.

## 5. Conclusion

The associations between adverse health issues and depressive symptoms as well as the association between poor cognitive function and depressive symptoms in older individuals with diabetes are worthy of further investigation. The findings from this study suggest that interventions targeted to help older adults properly manage their diabetes and reduce the likelihood of experiencing additional complications could possibly lead to a cost-effective option for healthcare professionals seeking the reduction of depressive symptoms. A common and low cost method for the reduction and management of distress in individuals living with diabetes seems to be education. Healthcare professionals can achieve this by providing the individual with knowledge regarding the health consequences of stress, teaching them various cognitive and behavioral skills to reduce physiological stress levels (e.g., recognition of major stressors in life, thought-stopping, and deep breathing), and educating them on better management of diabetes [35]. However, the extent to which lowered cognitive function influences the effectiveness of these educational programs in older adults with diabetes is not known.

Results from the current study may aid in the identification of older individuals living with diabetes who are at-risk for experiencing higher levels of depressive symptoms. The findings provide further evidence that having comorbid health issues may influence the presence of depressive symptoms. Given the higher rates of individuals living to older adulthood, many of whom are diagnosed with diabetes, interventions focused on diabetes distress could serve to aid a significant portion of the population in leading longer, healthier, and happier lives. These results are also important for clinicians and healthcare providers treating individuals with diabetes. These professionals should be educated about the myriad of issues and potential stressors that this population may face and how symptoms of distress and depression are manifested in order to ensure that proper treatment is given. While mood problems associated with diabetes may appear to be quite similar to those seen in clinical depression, healthcare providers and those living with diabetes alike should be aware of the possibility that these issues could be diabetes-specific and may need to be treated differently from the problems that are not unique to having diabetes. Given the interrelationship of cognitive impairment, depressive symptoms, and diabetes, further research is needed on intervention strategies for those with multiple problems in physical, cognitive, and psychological health.

## Figures and Tables

**Table 1:** Sample characteristics on study variables.

	Mean (SD)	*N* (%)	Sample range
Depressive Symptoms	2.73 (2.96)		0–14
Age	73.35 (6.09)		65–90
Education	13.50 (2.65)		2–20
Income	5.05 (1.95)		1–9
Female gender		129 (52.44)	
Married		117 (47.56)	
Race			
Caucasian		126 (51.22)	
African American		110 (44.72)	
Other		10 (4.07)	
Neuropathy		109 (44.31)	
Stroke		14 (5.69)	
Respiratory issues		52 (21.14)	
Arthritis		176 (71.54)	
Cardiac issues		58 (23.58)	
Cognitive function	23.37 (5.59)		8–39
Diabetes distress	3.85 (2.51)		2–12

**Table 2:** Correlations among depressive symptoms, demographic factors, health, and diabetes distress.

	(1)	(2)	(3)	(4)	(5)	(6)	(7)	(8)	(9)	(10)	(11)	(12)	(13)	(14)
(1) Depressive symptoms	1.00													

(2) Age	−.164 .010	1.00												

(3) Education	−.219 .001	.056 .383	1.00											

(4) Income	−.183 .004	−.093 .146	.529 <.001	1.00										

(5) Female gender	.047 .461	.164 .010	−.146 .022	−.389 <.001	1.00									

(6) Married	−.094 .141	−.133 .037	.100 .118	.473 <.001	−.397 <.001	1.00								

(7) African American race	.169 .008	−.141 .027	−.185 .004	−.383 <.001	.185 .004	−.234 <.001	1.00							

(8) Neuropathy	.210 <.001	.118 .066	−.057 .372	−.131 .039	.112 .079	−.161 .011	−.062 .336	1.00						

(9) Stroke	.356 <.001	−.176 .006	−.139 .029	−.006 .924	−.082 .199	.047 .462	−.009 .886	.099 .122	1.00					

(10) Respiratory issues	.200 .002	−.025 .702	−.015 .815	.013 .844	.054 .400	.065 .309	−.065 .309	.180 .005	.217 <.001	1.00				

(11) Arthritis	.174 .006	.091 .156	−.010 .874	−.058 .364	.229 <.001	−.103 .107	−.013 .843	.127 .046	.077 .228	.106 .100	1.00			

(12) Cardiac issues	.230 <.001	.054 .404	−.101 .113	−.132 .039	−.046 .470	.008 .901	−.076 .236	.102 .110	.194 .002	.135 .035	.032 .618	1.00		

(13) Cognitive function	−.266 <.001	−.066 .302	.323 <.001	.275 <.001	−.012 .852	.069 .282	−.324 <.001	−.060 .349	−.063 .323	−.027 .676	−.009 .888	−.043 .500	1.00	

(14) Diabetes distress	.598 <.001	−.122 .056	−.179 .005	−.227 <.001	.078 .225	−.133 .037	.190 .003	.157 .014	.127 .047	.062 .332	.100 .118	.170 .008	−.180 .005	1.00

**Table 3:** Covariate-adjusted associations between depressive symptoms and demographics, health, and diabetes distress.

	Model 1			Model 2			Model 3		
	*b*	SE	*B*	*b*	SE	*B*	*b*	SE	*B*
Age	−.08	.03	−.16^∗^	−.08	.03	−.16^∗∗^	−.05	.02	−.09
Education	−.17	.08	−.16^∗^	−.07	.08	−.07	−.04	.07	−.50
Income	−.09	.14	−.06	−.01	.12	−.01	−.05	.10	−.62
Female	−.07	.41	−.01	−.03	.38	.00	−.02	.32	−.94
Married	−.34	.44	−.06	−.35	.40	−.06	−.20	.33	−.03
Other races versus Caucasian	−.62	.95	−.04	−.57	.84	−.04	−1.35	.72	−.06
AA versus Caucasian	.45	.41	.08	.48	.39	.08	.11	.33	.02
Neuropathy				.82	.34	.14^∗^	.47	.29	.08
Stroke				3.03	.75	.24^∗∗∗^	2.79	.64	.22^∗∗∗^
Respiratory issues				.65	.42	.09	.61	.35	.08
Arthritis				.87	.38	.13^∗^	.59	.31	.09
Cardiac issues				1.06	.40	.15^∗∗^	.56	.34	.08
Cognitive function				−.10	.03	−.19^∗∗^	−.07	.03	−.14^∗∗^
Diabetes distress							.58	.06	.49^∗∗∗^

*Notes.* AA = African American; ^∗^ *p* < .05, ^∗∗^ *p* < .01, and ^∗∗∗^ *p* < .001.
